# EAST/SeSAME Syndrome and Beyond: The Spectrum of Kir4.1- and Kir5.1-Associated Channelopathies

**DOI:** 10.3389/fphys.2022.852674

**Published:** 2022-03-15

**Authors:** Jacky Lo, Anna-Lena Forst, Richard Warth, Anselm A. Zdebik

**Affiliations:** ^1^Neuroscience, Physiology and Pharmacology, University College London, London, United Kingdom; ^2^Medical Cell Biology, Institute of Physiology, University of Regensburg, Regensburg, Germany; ^3^Centre for Nephrology, University College London, London, United Kingdom

**Keywords:** KCNJ10, KCNJ16, EAST syndrome, SeSAME syndrome, channelopathy, epilepsy, deafness, distal convoluted tubule

## Abstract

In 2009, two groups independently linked human mutations in the inwardly rectifying K^+^ channel Kir4.1 (gene name *KCNJ10*) to a syndrome affecting the central nervous system (CNS), hearing, and renal tubular salt reabsorption. The autosomal recessive syndrome has been named EAST (epilepsy, ataxia, sensorineural deafness, and renal tubulopathy) or SeSAME syndrome (seizures, sensorineural deafness, ataxia, intellectual disability, and electrolyte imbalance), accordingly. Renal dysfunction in EAST/SeSAME patients results in loss of Na^+^, K^+^, and Mg^2+^ with urine, activation of the renin–angiotensin–aldosterone system, and hypokalemic metabolic alkalosis. Kir4.1 is highly expressed in affected organs: the CNS, inner ear, and kidney. In the kidney, it mostly forms heteromeric channels with Kir5.1 (*KCNJ16*). Biallelic loss-of-function mutations of Kir5.1 can also have disease significance, but the clinical symptoms differ substantially from those of EAST/SeSAME syndrome: although sensorineural hearing loss and hypokalemia are replicated, there is no alkalosis, but rather acidosis of variable severity; in contrast to EAST/SeSAME syndrome, the CNS is unaffected. This review provides a framework for understanding some of these differences and will guide the reader through the growing literature on Kir4.1 and Kir5.1, discussing the complex disease mechanisms and the variable expression of disease symptoms from a molecular and systems physiology perspective. Knowledge of the pathophysiology of these diseases and their multifaceted clinical spectrum is an important prerequisite for making the correct diagnosis and forms the basis for personalized therapies.

## Introduction

### K^+^ Channels: Membrane Proteins Important for Life

K^+^ channels are indispensable components in the mammalian organism, as they make a crucial contribution to the membrane potential and to electrical processes at the membrane. Therefore, defects of K^+^ channels lead to disturbances of these processes, possible consequences of which are altered neuronal excitability and hormone secretion as well as impaired membrane transport, cell volume regulation, and cell migration. The tasks of K^+^ channels in different cell types are unique and require specific functional and biophysical properties as well as a very precise coordination with other processes at the cell membrane and in the cell. In light of the manifold functional requirements, it is not surprising that the K^+^ channel family is the largest of the ion channels, comprising approximately 80 different pore-forming subunits.^1^^,^^2^ Hetero-oligomerization and splice variants further increase the diversity of different functional K^+^ channels and enable the organism to meet a wide variety of requirements. The NC-IUPHAR subcommittees on K^+^ channels have grouped channels based on gene family: Ca^2+^- and Na^+^-activated K^+^ channels (KCa, KNa); inwardly rectifying K^+^ channels (KIR); two-pore domain K^+^ channels (K2P); voltage-gated K^+^ channels (Kv), and their structure: channels that exhibit six, four or two transmembrane domains.^3^ This review article focuses on the pathophysiology of the inward rectifying K^+^ channels Kir4.1 and Kir5.1 and the diseases caused by mutations in their genes.

### Inward Rectifiers

Inwardly rectifying K^+^ channels have long been characterized functionally in the absence of detailed structural information (reviewed, for example, in Isomoto et al., 1997). Sir Bernard Katz noted a K^+^ current in muscle cells that increased with hyperpolarization and thus behaved fundamentally differently to the voltage-gated currents that increase with depolarization more commonly found in neurons (Katz, 1949). The first mammalian inward rectifier cloned is still widely known as Renal Outer Medullary K^+^ channel (ROMK) for its prominent expression in the renal thick ascending limb of Henle’s loop (TAL), distal convoluted tubule (DCT; Ho et al., 1993) and collecting duct (CD). Mutations in ROMK lead to Bartter syndrome (Bartter et al., 1962; Simon et al., 1996). This channel is also known as Kir1.1 or *KCNJ1* in genetic nomenclature and is the founding member of the inward rectifier family. The family has been grouped into seven classes, based on sequence homology, as outlined in (Doupnik et al., 1995), and now features subunits transcribed from 16 genes. Multiple splice products from the same gene, and heteromerization within, or, more rarely, across these seven classes, add considerable complexity. Many inward rectifiers are sensitive to one or multiple regulating factors, such as intracellular ATP/ADP concentration, membrane phosphatidylinositol 4,5-bisphosphate (PIP2), intracellular pH, phosphorylation, or are coupled to G protein-coupled receptors *via* trimeric G proteins, to name but a few. With channel crystallization came more detailed understanding of their functional mechanisms. The very first K^+^ channel crystalized, by Rod MacKinnon’s group (Doyle et al., 1998), was indeed an inward rectifier from Streptomyces lividans, now known as KcsA (PDB 1BL8). Like most other K^+^ channels, inward rectifiers are tetrameric and share structural similarity and a signature pore motif with most K^+^ channels known to date (Gonzalez et al., 2012). Inward rectification means that inward currents (K^+^ entering the cell) induced by the same absolute driving force (voltage difference between membrane voltage and K^+^ equilibrium potential) are greater than outward currents (K^+^ leaving the cell) driven in the opposite direction (Nichols and Lopatin, 1997). Detailed studies showed that this property depends on intracellular Mg^2+^ and polyamines partially blocking the pore at membrane voltages more depolarized than the K^+^ equilibrium potential (Fakler et al., 1995). Since K^+^ itself competes with these blockers for the pore, inwardly rectified currents often increase with increased extracellular K^+^ concentration despite a reduction in driving force, forming the physiological basis for K^+^-induced vasorelaxation in brain and muscle (Gonzalez et al., 2012). In contrast to most voltage-gated K^+^ channels commonly expressed in excitable cells, which are activated by depolarization, these channels exhibit larger conductance at more negative voltages and thus can stabilize the resting membrane voltage in non-excitable cells close to the K^+^ equilibrium potential. This is highly relevant to the Kir4-containing K^+^ channels discussed here, in the context of their function in glial cells and renal and stria vascularis epithelia.

## Molecular and Biophysical Properties of Kir4.1 and Kir5.1

### Cloning and Characterization of Kir4.1 (*KCNJ10*)

Kir4.1 was cloned independently by two groups (Bond et al., 1994; Takumi et al., 1995). Like other inward rectifiers (Hilgemann and Ball, 1996), its activity depends on PIP2 in the inner leaflet of the phospholipid bilayer and this has been studied even before structural information became available (Rapedius et al., 2007), building on research into other family members (Lopes et al., 2002; Schulze et al., 2003). Kir4.1-containing channels are also sensitive to intracellular pH, and in fact, the two properties may be closely linked (Rapedius et al., 2007). Loss-of-function mutations affecting PIP2 binding and regulation by intracellular pH in Kir4.1 highlight the importance of these regulatory mechanisms (Reichold et al., 2010). Whereas wild-type Kir4.1 activates around pH 6, Kir4.1^R65P^ and Kir4.1^R175Q^ (the latter also shows reduced PIP2 affinity) activate at intracellular pH values more alkaline than pH 7.5, which is unlikely to be reached in most cells. Since protons and PIP2 regulate the channel allosterically, there is scope for the development of small molecule activators with potential for personalized medicine.

### Heteromerization of Kir4.1 With Other Kir Subunits

**Figure 1:**
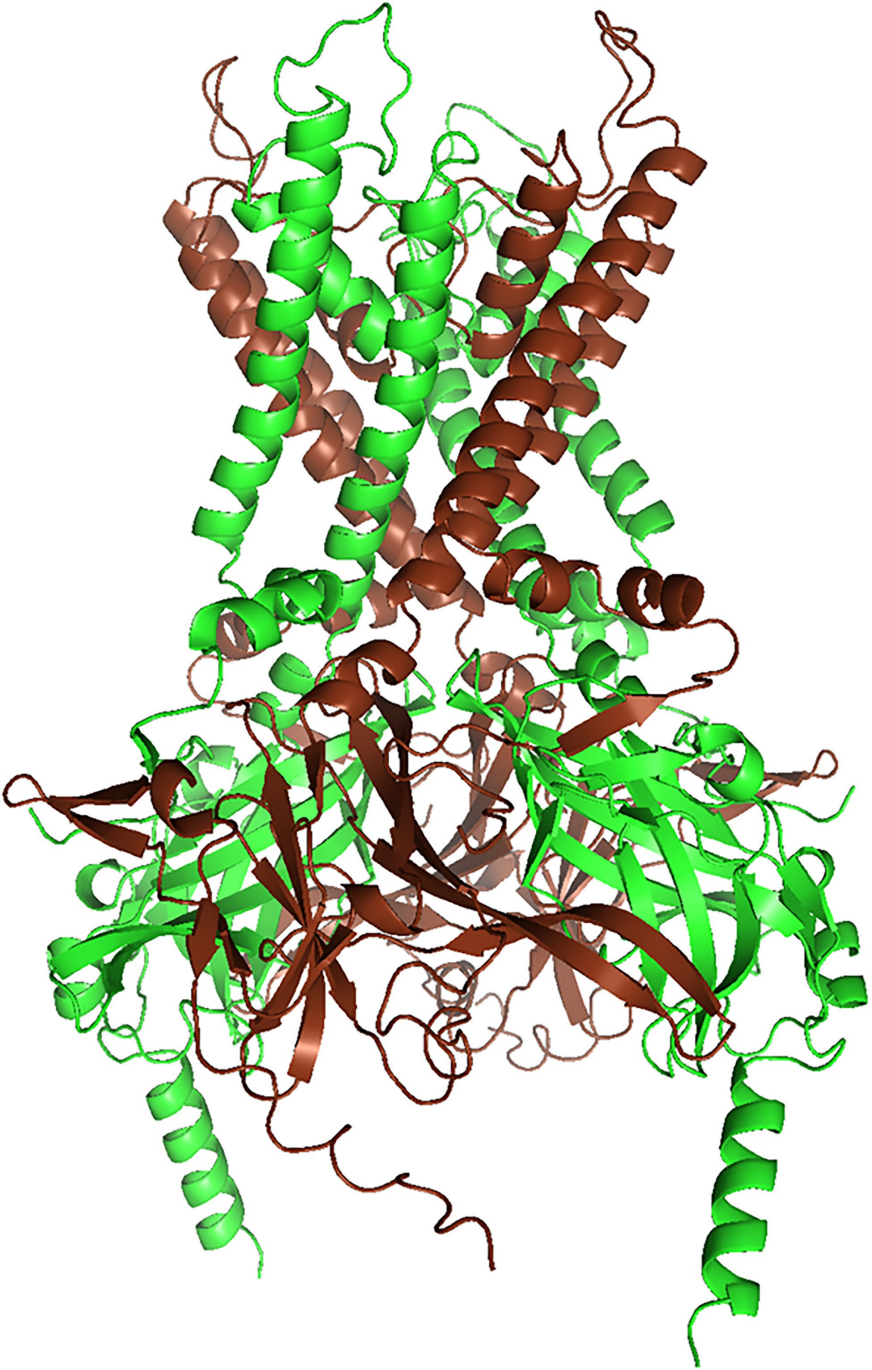
Structural model of the Kir4.1/Kir5.1 heteromer (rendered with PyMol 2.5.2) of a Kir4.1/Kir5.1 model generated with AlphaFold Multimer (Jumper et al., 2021, Evans and O’Neill, https://www.biorxiv.org/content/10.1101/2021.10.04.463034v1). We truncated both N- and C-termini slightly, to reduce complexity. AlphaFold yielded three models with almost identical ranking, but only one of them conformed to the alternating subunit structure postulated by Lagrutta et al. (1996). Kir4.1 subunits are brown, and Kir5.1 subunits are green.

Heteromerization of Kir4.1 with other subunits is more limited than for some other Kir family members. Kir4.1 forms weakly rectifying channels as homotetramer. As a heteromer with Kir5.1, it rectifies strongly (Lagrutta et al., 1996). By examining rectification of channels formed from concatenated subunits, Lagrutta et al. inferred that Kir4.1 and Kir5.1 alternate in the tetramer (Figure 1). Interestingly, heteromerization with Kir5.1 also shifts intracellular pH dependence of both Kir4.1 and Kir4.2 (which does not heteromerize with Kir4.1, but may compete with Kir4.1 for Kir5.1) into a more physiologically relevant pH range (Tucker et al., 2000; Pessia et al., 2001), and Kir4.1/Kir5.1 heteromeric channels may be involved in the pH-dependent regulation of respiration (Trapp et al., 2011): see section “Kir5.1 Variants: A Risk Factor for SIDS?”.

### Cloning and First Characterization of Kir5.1 (*KCNJ16*)

**Figure 2:**
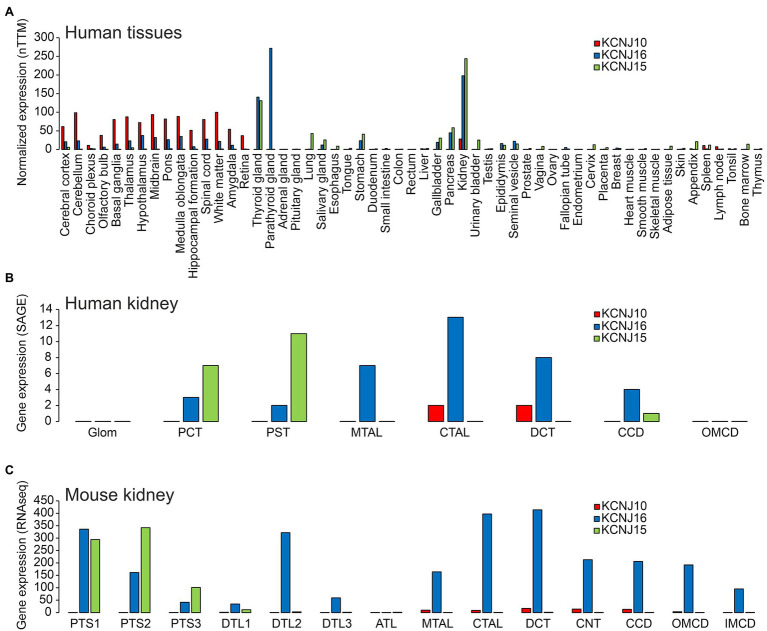
**(A)** Expression of KCNJ10 (Kir4.1), KCNJ16 (Kir5.1), and KCNJ15 (Kir4.2) in human tissues. Data were obtained from “The Human Protein Atlas” (https://www.proteinatlas.org/ENSG00000177807-KCNJ10/summary/rna; https://www.proteinatlas.org/ENSG00000153822-KCNJ16/summary/rna; https://www.proteinatlas.org/ENSG00000157551-KCNJ15/summary/rna). Data are presented as “consensus dataset” consisting of normalized expression (nTPM) levels, created by combining the HPA and GTEx transcriptomics datasets using the internal normalization pipeline of “The Human Protein Atlas” (https://www.proteinatlas.org). Please note that the number of samples for each tissue and the origin of the different tissues is different. Therefore, it can be difficult to quantitatively compare expression in different tissues. **(B)** Expression of KCNJ10, KCNJ16, and KCNJ15 along the human nephron. Data are taken from (Chabardes-Garonne et al., 2003). In this beautiful study, fresh human kidney tissue from nine donors was microdissected into glomeruli (glom), proximal convoluted tubules (PCT), proximal straight tubules (PST), medullary thick ascending limbs (MTAL), cortical thick ascending limbs (CTAL), distal convoluted tubules (DCT), cortical collecting ducts (CCD), and outer medullary collecting ducts (OMCD). Gene expression was assessed by “serial analysis of gene expression” (SAGE). **(C)** Expression of KCNJ10, KCNJ16, and KCNJ15 along the mouse tubular system. Data are taken from https://esbl.nhlbi.nih.gov/MRECA/Nephron/ (Chen et al., 2021). In this beautiful study, RNA-seq was performed on murine microdissected tubular segments. Initial segment of the proximal tubule (PTS1); proximal straight tubule in cortical medullary rays (PTS2); last segment of the proximal straight tubule in the outer stripe of outer medulla (PTS3); short descending limb of the loop of Henle (DTL1); long descending limb of the loop of Henle in the outer medulla (DTL2); long descending limb of the loop of Henle in the inner medulla (DTL3); thin ascending limb of the loop of Henle (ATL); medullary thick ascending limb of the loop of Henle (MTAL); cortical thick ascending limb of the loop of Henle (CTAL); macula densa (MD); distal convoluted tubule (DCT); connecting tubule (CNT); cortical collecting duct (CCD); outer medullary collecting duct (OMCD); inner medullary collecting duct (IMCD). Another excellent source of transcriptome data is https://cello.shinyapps.io/kidneycellexplorer/, which provides single cell RNA-seq data of mouse kidney (Ransick et al., 2019). These single cell-based data indicate that Kir4.1 and Kir5.1 are strongly expressed in principal cells of connecting tubules and collecting ducts, but not in intercalated cells (data not shown).

Kir5.1 was first described in a conference abstract (Bond et al., 1994) and subsequently characterized in more detail (Pessia et al., 1996). These initial studies (corroborated by most subsequent work) concluded that Kir5.1 cannot form functional channels when expressed in oocytes alone, but that it modified current properties of Kir4.1 when coexpressed. Heteromeric channels produced currents that were bigger, more strongly rectified and which also showed some time dependence in comparison to homotetrameric Kir4.1. Tanemoto et al. (2002) noticed a PDZ binding motif in Kir5.1 and observed inwardly rectified currents when coexpressing PSD-95 in HEK cells. Moreover, Kir5.1 expressed alone showed diffuse cytoplasmic localization, whereas PSD-95 coexpression led to clustering of Kir5.1 at the plasma membrane, suggesting that Kir5.1 could have a role in the postsynaptic terminal. The relatively high expression level of Kir5.1 in some organs (Figure 2) in the absence of Kir4.1 could suggest that Kir5.1-mediated currents depend on anchoring proteins, other than heteromerization with Kir4.1 or Kir4.2. However, the expression pattern of Kir4.2 has not been as firmly established as good antibodies are lacking, so an alternative explanation for functional expression of Kir5.1 could be heteromerization with other channel subunits, possibly even Kir2.1 (Fakler et al., 1996). However, the genetic evidence discussed below, with the absence of CNS symptoms in patients with homozygous or compound heterozygous Kir5.1 mutations at least makes this possibility less likely, as all Kir5.1 mutations investigated, scattered over different functional domains of Kir5.1, strongly affected heteromers with either Kir4.1 or Kir4.2 (Schlingmann et al., 2021).

### Tissue-Specific Expression

The expression of Kir4.1, Kir5.1, and other inward rectifying channels is highly variable and specific in different tissues of the organism. Understanding this, and thus, the possibility of forming heteromeric channels is key to predicting which organs may be affected by channel mutations and which clinical symptoms may result. Current research suggests that the symptomatology triggered by Kir4.1 mutations is primarily due to disruption of Kir4.1 homomers and Kir4.1/Kir5.1 heteromers. Unlike Kir4.1, Kir5.1 does not appear to form homomeric channels, but forms heteromeric channels primarily with Kir4.1 and Kir4.2. For this reason, Kir4.2 was also included in the representation of tissue expression (Figure 2) in addition to Kir4.1 and Kir5.1. However, it can be assumed that in addition to these heteromers highlighted here, others exist with other members of the Kir family. Kir4.1 shows highest levels of expression in various brain regions (Figure 2A). Kir4.1, and to a much lesser degree Kir5.1, is expressed predominantly in glial cells, most highly at astrocyte end feet, which are in close contact to cerebral blood vessels (Poopalasundaram et al., 2000), but some expression of Kir4.1 was also evident at astrocyte processes close to synaptic clefts (Higashi et al., 2001). In some brain regions, homomeric Kir4.1 channels are expressed, whereas heteromeric channels with Kir5.1 are found in others (Hibino et al., 2004a). Immunohistochemistry confirmed expression of Kir4.1 in astrocytes throughout the brain (Li et al., 2001; Hibino et al., 2004a). Using both immunoprecipitation and immunohistochemical methods, the latter group identified homomeric Kir4.1 or Kir4.1/Kir5.1 heteromers in astrocyte endfeet facing blood vessels. In peripheral tissues, the highest Kir4.1 expression levels are found in the kidney (Takumi et al., 1995; Lourdel et al., 2002; Lachheb et al., 2008; Bockenhauer et al., 2009; Reichold et al., 2010). Throughout the tubular system, Kir4.1 is found in the basolateral membrane of only a few segments, that is, the thick ascending limb of Henle (TAL), the distal convoluted tubule (DCT), and in the principal cells of the connecting tubule (CNT) and cortical collecting duct (CCD; Figures 2B,C). Kir5.1 is also expressed in several brain regions, where it may underlie pH-sensitive K^+^ conductance. In peripheral tissues, Kir5.1 forms heteromeric channels with Kir4.1 mainly in the tubular segments of the kidney, where Kir4.1 is expressed. In other nephron segments, thyroid, and gastric parietal cells (Lambrecht et al., 2005), Kir5.1 likely forms heteromeric channels with Kir4.2 and/or other Kir channels (Figure 2A). Kir4.2 is most highly expressed in renal proximal tubules, where it likely assembles with Kir5.1 to form basolateral K^+^ channels (Bignon et al., 2020; Figures 2A–C). In addition to the tissues shown in Figure 2, Kir4.1 and Kir5.1 are also expressed in the inner ear. The expression will be discussed in more detail in the sections on hearing impairment with Kir4.1 and Kir5.1 mutations.

## EAST/SeSAME Syndrome: A Pleiotropic Monogenetic Disease Caused by Mutations in *KCNJ10* (Kir4.1)

This syndromic disease affects hearing, the kidney, and CNS. Mutations in Kir4.1 typically affect the function of both homomeric Kir4.1 (Bockenhauer et al., 2009) as well as heteromeric Kir4.1/Kir5.1 (Reichold et al., 2010) channels, although there are some interesting exceptions to this rule (see section “Autism,” Table 1). EAST is an acronym for epilepsy, ataxia, sensorineural hearing loss, and renal tubulopathy (Bockenhauer et al., 2009). It is also known as SeSAME syndrome (seizures, sensorineural deafness, ataxia, intellectual disability, and electrolyte imbalance; Scholl et al., 2009).

**Table 1:** Mutations identified in *KCNJ10*/Kir4.1.

Mutation	CADD score	Associated phenotypes					Typical/Atypical/ASD	Function (expressed alone)	Reference
		E	A	S	T	WM			
R18Q	22.4	Y	Y	N	N	N	ASD	Gain of function	Sicca et al., 2011, 2016
T57I	25.8	Y	Y	Y	Y	N	T	Complete LOF	Scholl et al., 2012
I60T	24.9	Y	Y	ND	N	Y	A	Not determined	Al Dhaibani et al., 2018
I60M	22.5	N	Y	Y	N	N	A	Not determined	Nicita et al., 2018
R65C	29.1	Y	Y	Y	Y	N	T	Not determined	Papavasiliou et al., 2017
R65C	29.1	Y	Y	Y	Y	Y	T	Not determined	Celmina et al., 2019
R65C	29.1	Y	Y	Y	Y	ND	T	82% reduction in currents	Freudenthal et al., 2011
R65P	28.5	Y	ND	ND	ND	ND	T	Not determined	Thompson et al., 2011
R65P	28.5	Y	Y	Y	Y	N	T	75% reduction in currents	Bockenhauer et al., 2009; Williams et al., 2010
R65P	28.5	Y	Y	Y	Y	ND	T	>80% reduction in currents	Scholl et al., 2009; Reichold et al., 2010; Williams et al., 2010
L68P	26.0	Y	Y	?	?	?	?	Not determined	Lemke et al., 2012
F75C	27.7	Y	Y	Y	Y	ND	T	Complete loss of function	Parrock et al., 2013
F75L	23.6	Y	Y	Y	Y	ND	T	>90% reduction in currents	Freudenthal et al., 2011
G77R	25.2	Y	Y	Y	Y	ND	T	>90% reduction in currents	Bockenhauer et al., 2009; Reichold et al., 2010; Williams et al., 2010
G83V	26.4	?	?	?	?	?	?	Complete loss of function	Mendez-Gonzalez et al., 2016
V84M	24.9	Y	N	N	N	N	ASD	Gain of function	Sicca et al., 2011
V91Gfs*197		Y	Y	Y	Y	ND	T	Complete loss of function	Parrock et al., 2013
F119Gfs*25		Y	Y	Y	Y	N	T	Not determined	Papavasiliou et al., 2017
I129V	24.6	Y	Y	?	?	?	?	Not determined	Lemke et al., 2012
C140R	26.7	Y	Y	Y	Y	ND	T	Complete loss of function	Scholl et al., 2009; Williams et al., 2010
G163D (1)	26.8	N	Y	Y	N	N	A	Complete loss of function	Morin et al., 2020
T164I	23.6	Y	Y	Y	Y	Y	T	Complete loss of function	Scholl et al., 2009; Williams et al., 2010
L166Q	27.2	?	?	?	?	?	?	50% reduction in currents	Mendez-Gonzalez et al., 2016
A167V	25.8	N	N	N	Y	ND	A	40% reduction in currents	Parrock et al., 2013; Suzumoto et al., 2021
A167V (6)	25.8	Y	Y	Y	Y	ND	T	~50% with R297C	Scholl et al., 2009; Williams et al., 2010
R171Q (1)	28.1	N	Y	Y	N	N	A	50% reduction in currents	Morin et al., 2020
R175Q	29.8	Y	Y	Y	Y	ND	T	>90% reduction in currents	Reichold et al., 2010
P194H (2)	23.7	N	N	Y	N	ND	A	51% reduction in currents	Yang et al., 2009
R199*		Y	Y	Y	Y	ND	?	Complete loss of function	Scholl et al., 2009; Reichold et al., 2010; Williams et al., 2010; Thompson et al., 2011
A201T (3)	27.4	Y	Y	N	N	N	A	Almost complete loss of function	Zhang et al., 2019
R204H	29.2	Y	Y	Y	Y	N	T	Not determined	Kara et al., 2013
I209T (3)	25.9	Y	Y	N	N	N	A	37% reduction in currents	Zhang et al., 2019
Q212R	26.0	?	?	?	?	?	?	Currents similar to WT	Mendez-Gonzalez et al., 2016
L218F (4)	27.3	Y	Y	N	N	N	A	60% reduction in currents	Hasan et al., 2017
N232Qfs*14		Y	Y	Y	Y	Y	T	Not determined	Severino et al., 2018; Suzumoto et al., 2021
V259*		Y	Y	Y	Y	ND	T	Complete loss of function	Freudenthal et al., 2011
G275Vfs*7		Y	Y	N	Y	Y	A	Predicted to be deleterious	Severino et al., 2018; Suzumoto et al., 2021
T290A	25.8	Y	Y	Y	N	N	A	60% reduced currents (LCL)	Nadella et al., 2019
R297C	32	Y	Y	Y	Y	ND	T	>90% reduction in currents	Freudenthal et al., 2011; Thompson et al., 2011
R297C	32	Y	Y	Y	Y	ND	T	Complete loss of function	Scholl et al., 2009; Williams et al., 2010
R348C (2)	22.7	N	N	Y	N	ND	?	44% reduction in currents	Yang et al., 2009
R348H	16.5	Y	N	N	N	ND	ASD	Gain of function	Sicca et al., 2016
		>25% Residual function							
		<25% Residual function							
		Atypical features							
		Gain of function							
		Function unknown							

Table 1 summarizes the consequences of KCNJ10 mutations. CADD score according to https://cadd.gs.washington.edu/snv (Rentzsch et al., 2019). Associated phenotypes refer to epilepsy (E), ataxia (A), sensorineural deafness (S), tubulopathy, hypokalemia and alkalosis (T), white matter abnormalities (WM), and autism spectrum disorder (ASD). Yes (Y); No (N); Not determined (ND). * indicates a “stop codon”. Color code is shown for functional effects when expressed as homomeric Kir4.1. Note that pink coloration was given to “atypical” (=A) mutations which lead to partial EAST syndrome, that is, lack some of its characteristic features, as opposed to “typical,” featuring all cardinal symptoms Epilepsy, Ataxia, Sensorineural hearing loss, and renal Tubulopathy. LCL: Kir4.1 T290A was examined in patient-derived lymphoblast cells. (1) Compound heterozygous state. (2) With mutations in Slc26A4. (3) Compound heterozygous state. (4) With mutation in KCNT1. (5) With mutations in Slc26A4.

We will start by describing the typical symptoms seen in most patients carrying loss-of-function mutations in Kir4.1, affecting both homomeric as well as Kir4.1/Kir5.1 heteromers.

### Epilepsy

**Figure 3:**
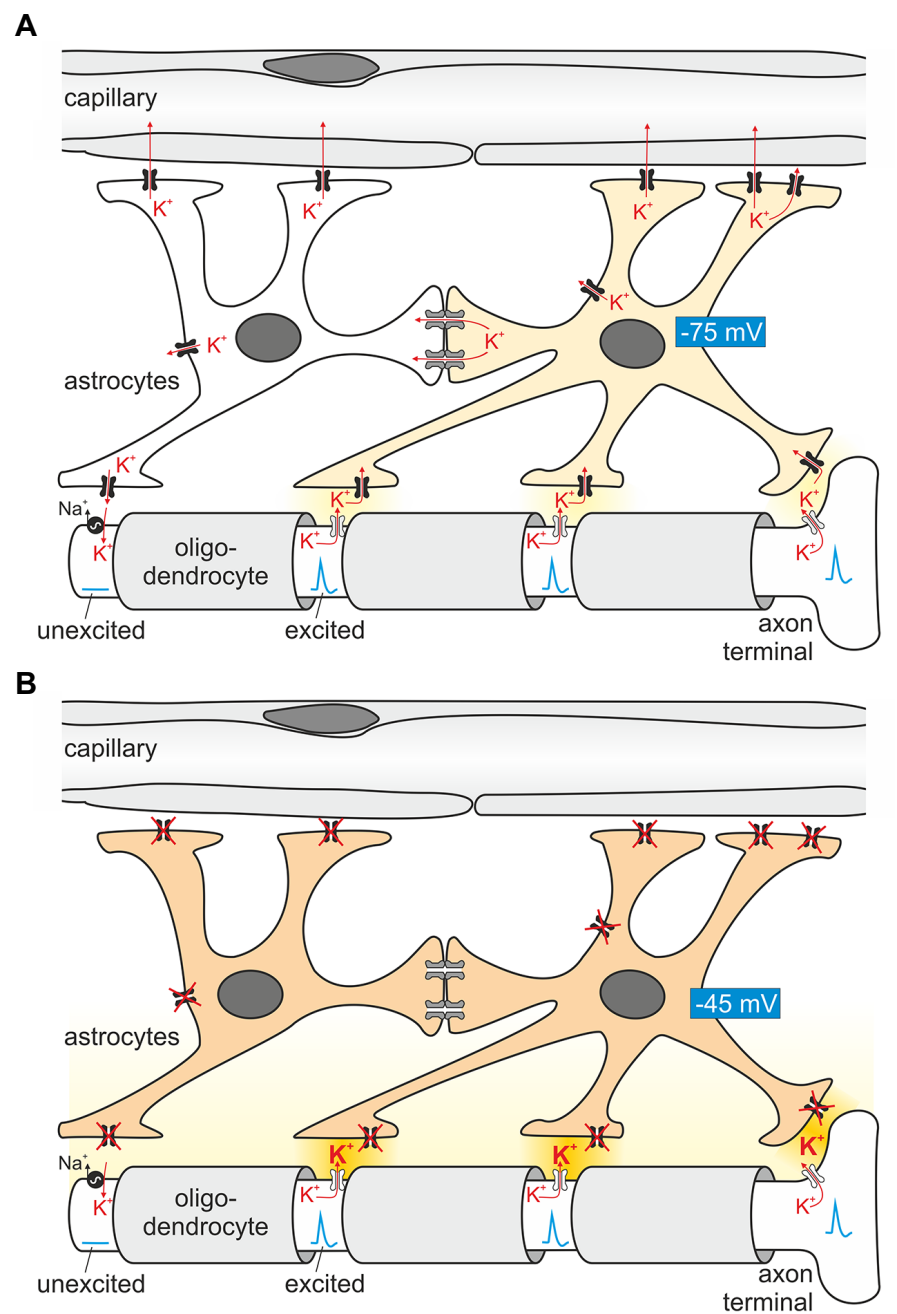
Principles of astroglial K^+^ buffering. **(A)** Astrocytes are in close contact with Ranvier nodes, exposed parts of the axon between segments wrapped with myelin (light gray) provided by oligodendrocytes. The nodes “regenerate” the action potential as it travels in a saltatory (jumping) mode to the next node. Conduction between nodes is electrotonic. When firing, Na^+^ enters the axon, depolarizes the nodal axolemma and K^+^ is leaving the axon through K^+^ channels (Kanda et al., 2019). The ensuing K^+^ exit from the axon increases extracellular K^+^ locally and induces K^+^ uptake by astrocyte processes in close proximity. A prerequisite of locally restricted K^+^ uptake by astrocytes is their hyperpolarized membrane voltage (requiring Kir4.1 channels depicted in black). On the other hand, K^+^ uptake depolarizes the astrocyte membrane and enhances the driving force for K^+^ exit at areas closer to blood vessels, where the K^+^ concentration is low. In addition, K^+^ is also transferred by electrical coupling to neighboring astrocytes which are, at this moment in time, not surrounded by increased K^+^. Once the nodal axon repolarizes, the axolemmal Na^+^/K^+^-ATPase takes up K^+^ released slowly by the astrocyte. **(B)** When astrocytes lack functional Kir4.1 channels, extracellular K^+^ is expected to rise (darker yellow extracellular space), and spontaneous action potential generation ensues, explaining the epilepsy seen in EAST patients.

Epileptic seizures occur in ~0.5% of the population over their lifetime (Schroeder et al., 1998), and monogenic causes include a wide variety of proteins (Guerrini et al., 2021). Some of the first genes identified in epilepsy families encode for ion channels. They include mutations in subunits of Na^+^ channels (Escayg et al., 2000; Vanoye et al., 2014) and K^+^ channels (Biervert et al., 1998; Schroeder et al., 1998). These channels are expressed in neurons and directly affect neuronal excitability. KCNQ2/KCNQ3, for example, mediate the important “M current”, named after its characteristic inhibition by muscarinic stimulation (Brown and Adams, 1980). At that time, research into epilepsy was decidedly focused on neurons. However, Kir4.1, and to a much lesser degree Kir5.1, are expressed predominantly in glial cells. Long before the first K^+^ channels were cloned, it was speculated that glial cells were involved in spatial K^+^ buffering (Kuffler et al., 1966; Orkand et al., 1966). This concept postulates that glial cells use K^+^ channels and other K^+^ transporting membrane proteins to funnel K^+^ away from sites of high neuronal activity (where the local extracellular K^+^ concentration is high enough for entry into glial cells) toward blood vessels, where the extracellular K^+^ concentration is low. Figure 3 illustrates how K^+^ handling occurs at the nodes of Ranvier. They are instrumental for regenerating the action potential using voltage-gated Na^+^ channels, as excitation jumps electrotonically from node to node. In the CNS, voltage-gated K^+^ channels (Kv) predominate in the paranode for repolarization, but in the peripheral nervous system there are “leak” K^+^ channels in addition, expressed in the nodal axon itself (Kanda et al., 2019). Astrocytes use Kir4.1 channels to temporarily absorb K^+^ released at the junctions between oligodendrocytes where the action potential is actively regenerated. It is noteworthy that Kir channels are uniquely suited for this task as their conductance will increase when extracellular K^+^ concentration rises. In addition, homomeric Kir4.1 is less rectifying than some other members of the family, which might facilitate movement of K^+^ in both directions. Butt and Kalsi (2006) summarize the spatial buffering concept. Electrical coupling of astrocytes by gap junctions may help to extend their reach and K^+^ buffer capacity. It may also help to maintain their hyperpolarized membrane voltage at sites of neuronal K^+^ release, allowing the unusual passive entry of K^+^ into the astrocyte. Since K^+^ is lower in the perivascular space, astrocytes may release their K^+^ at their endfeet contacting vessels. In support of the role of electrical coupling, it is known that gap junctions are more numerous in gray matter astrocytes, where more K^+^ is released. Indeed, K^+^ clearance from the extracellular space was shown to be dependent on astrocytic gap junctions (Wallraff et al., 2006). Seizures as a major symptom of loss of function in homomeric Kir4.1 (Bockenhauer et al., 2009; Scholl et al., 2009) suggest that Kir4.1 channels are crucial for human brain K^+^ homeostasis. More direct, functional evidence for a major role for Kir4.1-containing channels for removal of K^+^ from the synaptic cleft came from studies in astrocyte-specific Kir4.1 knockout mice (Djukic et al., 2007), which showed depolarized astrocytes and complex glia, as well as changes to synaptic potentiation, suggesting that the change in astrocyte function can have long-term effects on synaptic transmission. In addition, K^+^ clearance capability correlated with Kir4.1 expression level in the spinal cord (Olsen et al., 2007). More recently, K^+^ clearance following tetanic stimulation was directly shown to be delayed in hippocampal slices from glial-specific conditional Kir4.1 knockout mice using ion-selective microelectrodes, although resting K^+^ was unchanged (Haj-Yasein et al., 2011). The latter may be a result of testing this parameter in slices, rather than intact tissue, where diffusion in the extracellular space may be more limited. Interestingly, this group did not observe any evidence for osmotic changes on stimulating wild-type or Kir4.1-deficient slices, which suggests that K^+^ movement is accompanied by movement of water. Indeed, AQP-4 was shown to be present in astrocyte endfeet (Neely et al., 2001; Hibino et al., 2004a) and may even form a protein complex with other homoeostatic proteins (AQP4 and the dystrophin complex; Horio et al., 1997; Neely et al., 2001), mutations of which have been shown to cause white matter changes (see section “Is There Structural Change to the CNS? The MLC1 Connection in Oligodendrocytes”). Altered expression/localization of Kir4.1 channels has been found in tissue from pharmacoresistant seizure patients. Loss of its expression has also been reported for sclerotic hippocampal tissue from mesial temporal lobe epilepsy patients (Heuser et al., 2012), but these could reflect secondary changes. However, the important role of glial Kir4.1 for K^+^ clearance *per se* is well established, and loss of Kir4.1 function causes epilepsy not only in humans (Bockenhauer et al., 2009), but also mice (Djukic et al., 2007), dogs (Gilliam et al., 2014), and zebrafish (Bockenhauer et al., 2009).

### Ataxia

Bergmann glial cells, as well as Müller cells in the retina, are radial glial cells which are hugely important as a scaffold for migrating neurons in the developing cerebral and cerebellar cortex as well as the retina. They exclusively persist in the adult cerebellum and retina and continue to have important roles in maintaining the integrity of these structures (Mori et al., 2005). Cerebellar Purkinje cells have two distinct “resting” membrane voltage states and display a relatively high “resting” spontaneous firing rate, with much higher frequencies when they are in the more depolarized state (Hausser et al., 2004). Bergmann glial cells are thought to modify Purkinje cell spiking *via* regulating K^+^ homeostasis (Wang et al., 2012). Their close proximity to Purkinje cells and the high expression of Kir4.1 suggests that Kir4.1 might be involved in this function. Many patients with homozygous or compound heterozygous loss-of-function mutations in Kir4.1 suffer from severe, debilitating ataxia from infancy. They showed pronounced gait ataxia, intention tremor, and dysdiadochokinesis (Bockenhauer et al., 2009), with some affected individuals being unable to walk. Meanwhile, disruptive mutations have been identified in older atactic patients (Morin et al., 2020), and cerebellar imaging has revealed progressive cerebellar vermis degeneration in some of these individuals, while more discrete changes in MRI seem to prevail at younger age (Scholl et al., 2009), which were not appreciated in our early study (Bockenhauer et al., 2009), nor in a clinical update on the initial study (Abdelhadi et al., 2016). The reported signs and symptoms in children clearly indicate cerebellar dysfunction. However, it remains to be determined whether ataxia originates from subtle developmental effects, functional defects in K^+^ homeostasis, degenerative processes, or, most likely, a combination thereof. Structural change of the cerebral cortex will be discussed below in the context of white matter abnormalities.

### Retinal Expression and Electroretinographic Changes

Müller cells are a type of radial glial cell specific to and persisting throughout life in the retina, which also highly expresses Kir4.1 (Ishii et al., 1997; Kofuji et al., 2000), particularly in its endfeet membranes (Kofuji et al., 2002). However, no defects in vision have been reported in patients to date. EAST patients do exhibit electroretinographic changes, some of which are likely due to impaired K^+^ homoeostasis and others secondary to reduced glutamate retrieval by Müller cells, which depends on the K^+^-dependent Müller cell membrane potential (Thompson et al., 2011). Interestingly, Müller cells were shown to express both Kir4.1 homomeric as well as (presumably) Kir4.1 and 5.1 heteromeric channels. Photoreceptors are highly active metabolically, producing lactic acid, which could induce a pH gradient across the retina. Whether this arrangement of pure Kir4.1 at the endfeet and heteromeric Kir4.1/5.1 elsewhere allows Müller cells to maintain their K^+^ conductance, or even make it respond to demand, is currently unclear, as is the significance of loss of Kir4.1 function for the retina.

### Intellectual Disability

Before we begin discussing intellectual disability, it is worth noting that due to the severity of other neurological symptoms, children with EAST syndrome are actually difficult to examine (Abdelhadi et al., 2016). Severe ataxia and sensorineural hearing loss are likely contributing to slowed speech and impaired general intellectual development, particularly in early years. Epileptic seizures may lead to excitotoxicity, in turn causing intellectual disability, which may otherwise be absent. Conversely, learning and intellectual ability also have morphological correlates, such as changes to dendrite morphology, albeit more discreet than currently detectable by standard imaging techniques. It is clear, however, that with optimal treatment some children with functionally disruptive mutations in KCNJ10 manage to attend normal schools (Abdelhadi et al., 2016), particularly if symptoms are picked up early, suggesting that intellectual disability could be secondary to the other forms of disability experienced by these patients.

### Is there Structural Change to the CNS? The MLC1 Connection in Oligodendrocytes

K^+^ channels may have an interesting additional role in oligodendrocyte precursor cells, which were shown to upregulate their Kir4.1 currents and mRNA expression during maturation (Maldonado et al., 2013). Following up from (Scholl et al., 2009), who reported spinal cord abnormalities as well as peripheral nerve abnormalities in children with SeSAME syndrome, a longitudinal study has concluded that there are subtle and variable morphological changes both to cerebellar nuclei as well as white matter structures in the CNS (Cross et al., 2013). The white matter changes were most pronounced in the corpus callosum, which predominantly mediates communication between neocortical hemispheres, and appeared stable. However, the authors suggested that more longitudinal studies in older patients were required to settle this question. The MRI signal abnormalities reported could be consistent with some vacuolization of myelin, which has been observed in Kir4.1 KO mice (Neusch et al., 2001). In this work, which focused on the spinal cord of young mice (up to ~day 20, due to early mortality in KO mice), it is mostly oligodendrocytes which appear to be affected, and this matches with immunohistochemical localization of Kir4.1 in these young mice, which, in this study, was exclusive to oligodendrocytes. The vacuolization observed, although more discreet, shows some parallels with both humans and mice deficient in MLC1, a protein of still somewhat unclear function (reviewed in Bosch and Estevez, 2020). It is believed to establish a complex with other oligodendrocyte membrane proteins (Lanciotti et al., 2012). These proteins include Kir4.1, GlialCAM, and AQP-4. Independently, GlialCAM was shown to dramatically alter the gating of ClC-2, a Cl^−^ channel, which changes its strong inward rectification to a “leak-like,” more linear conductance upon coexpression with GlialCAM (Jeworutzki et al., 2012). Together, they could furnish developing oligodendrocytes with a mechanism to reduce their cytoplasmic volume while wrapping around axons, satisfying charge neutrality (parallel Cl^−^ flux through ClC-2) and isoosmolarity (owing to AQP4). It is predicted that failure to do so would result in vacuoles or less compact myelin sheaths, which was described for Kir4.1 knockout mice (Neusch et al., 2001), and has also be observed in ClC-2 knockout mice. It is possible that this phenotype is less pronounced in humans because myelinization occurs over a much longer time scale. Alternatively, astrocytic expression could have independent and stronger effects in humans on other phenotypes, mediated by disrupted function of astrocytes and Bergmann glial cells, as outlined above. In any case, we are likely to learn from vacuolization in other mouse models, and possibly human postmortem tissue, in the future.

### Sensorineural Deafness

The inner ear hair cells are unusual in their handling of K^+^. While most cells stabilize their membrane voltage using K^+^ channels and the steep K^+^ gradient generated by Na^+^/K^+^-ATPases (Skou, 1957), the specific arrangement of inner ear hair cells allows K^+^ to both enter and leave the cell passively. This may serve to compartmentalize blood flow and vibration blood flow entails away from the highly sensitive hair cells. This is probably crucial to achieve the high dynamic range of hearing, which covers nearly six orders of magnitude in sound pressure level. The stria vascularis, the only human epithelium with intraepithelial vessels, generates the driving force for this passive K^+^ movement across the hair cells. It provides both a high extracellular K^+^ concentration as well as a positive voltage, the endocochlear potential, to the endolymph [reviewed, for example, in (Zdebik et al., 2009)], which is in contact with the apical side of the hair cell. Its insulation from the perilymph, more similar in ionic composition to extracellular fluid found elsewhere in the human body, and in contact with the basal part of the hair cells, is notably dependent on the expression of certain “sealing” claudins, Claudin-14 and Claudin-9, and disruption of these claudins in mice, or mutations in humans also lead to deafness (Wilcox et al., 2001; Nakano et al., 2009). Kir4.1 is particularly crucial for the endocochlear potential, as it is largely generated across the luminal membrane of strial intermediate cells which show high expression of Kir4.1 (Marcus et al., 2002). Efficient removal of K^+^ from the intrastrial apical fluid facing the strial intermediate cells is also crucial, shown by loss of endocochlear potential and hearing in barttin knockout mice (Rickheit et al., 2008), compromising chloride recycling across strial marginal cells by abolishing function of both ClC-Ka and ClC-Kb. There is also hearing loss in humans with mutations in both ClC-Ka and Kb channels (Schlingmann et al., 2004). Electrolyte abnormalities in the endolymph may also affect the survival of outer hair cells which have been shown to degenerate in several mouse models with targeted disruption of inner ear K^+^ recycling mechanisms [reviewed in (Zdebik et al., 2009)]. Lastly, loss of expression of Kir4.1 has been shown to underlie hearing loss in mice with targeted disruption of pendrin, an anion transporter also important for iodination in the thyroid (Wangemann et al., 2004). Heterozygous human mutations in pendrin are usually benign, but when paired with a heterozygous mutation in Kir4.1 may also lead to deafness with digenic inheritance (Yang et al., 2009).

### Autism

Several reports from the same group have described dominant, gain-of-function mutations in Kir4.1 associated with Autism Spectrum Disorder (Sicca et al., 2016), reviewed in Cheng et al. (2021). These mutations have shown mild gain of function, due to several mechanisms (increased surface expression, open probability and single channel conductance; Sicca et al., 2016). Interestingly, they also described epilepsy in these patients. While two of these mutations are located in parts of Kir4.1 that might affect trafficking and stability, it is currently unclear how they affect single channel conductance. Even more unclear is how gain-of-function mutations in K^+^ channels can lead to epilepsy. Further research is needed to place these mutations in the context of all other mutations which invariably lead to loss of function alone or when coexpressed with Kir5.1.

### Renal Tubulopathy

Tubular transport processes in the kidneys are central to the water and electrolyte homeostasis of the body. In this context, the function of K^+^ channels is pivotal for numerous transport processes by hyperpolarizing the membrane potential and providing the energy for potential-dependent transport (Warth, 2003). In addition, apically located K^+^ channels provide a pathway for direct K^+^ secretion, allowing K^+^ to be efficiently and rapidly excreted in the urine upon an increase in plasma K^+^ (Hebert et al., 2005). While the proximal tubule and thick ascending limb of Henle’s loop provide the bulk reabsorption, it is the task of the distal nephron to precisely match urine volume and composition to the body’s particular requirements. To allow accurate adjustment, transepithelial transport processes including K^+^ conductances in the distal nephron are precisely controlled and fine-tuned by various hormones, mediators—and in the case of urinary K^+^ excretion—also by plasma K^+^ itself.

**Figure 4:**
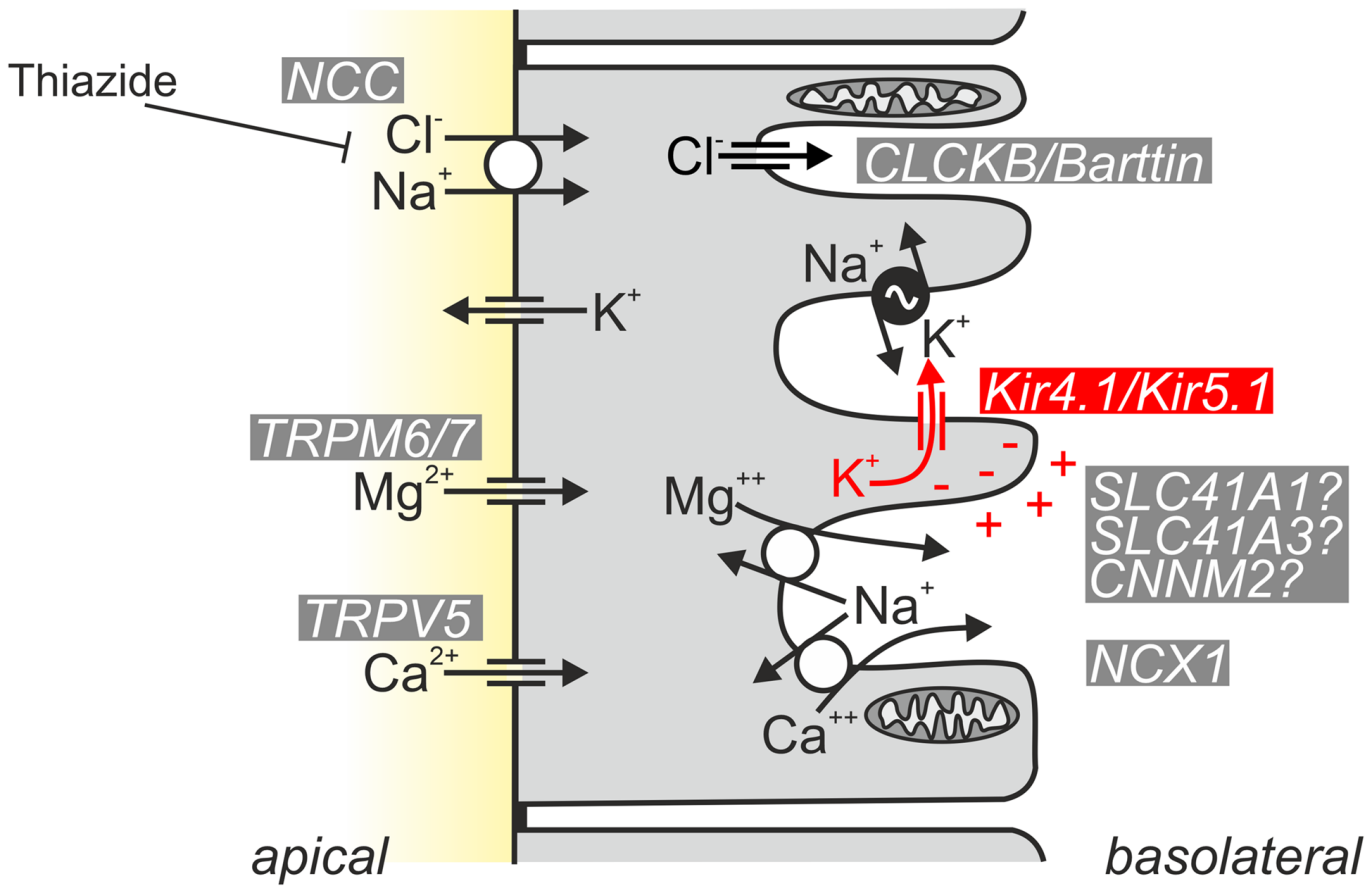
Schematic of normal Kir4.1/Kir5.1 function in the DCT. Kir4.1/Kir5.1 heteromeric channels are localized in the basolateral membrane, which has numerous deep infoldings. There, the channel ensures that sufficient K^+^ is available to be taken up by the Na^+^/K^+^-ATPase (pump–leak coupling). Moreover, Kir4.1/Kir5.1 channels hyperpolarize the basolateral membrane, generating the driving force for the potential-dependent export of Mg^2+^ and Ca^2+^ and the efflux of Cl^−^ through CLCKB/Barttin Cl^−^ channels. The latter has implications for cytosolic Cl^−^ concentration and is thought to indirectly modulate NCC activity. Ca^2+^ is also extruded basolaterally *via* Ca^2+^-ATPase (not shown). The molecular nature of the pathway for basolateral Mg^2+^ extrusion is still a matter of debate; likely candidates are shown.

What is the renal phenotype in classical EAST/SeSAME syndrome? The main symptoms are metabolic alkalosis, hypokalemia, and hypomagnesemia. Hypocalciuria increased urinary Na^+^ loss, and increased plasma renin and aldosterone concentrations are also observed (Bockenhauer et al., 2009; Scholl et al., 2009). The tubulopathy and resulting electrolyte disturbance of EAST/SeSAME patients are reminiscent of the symptoms in Gitelman syndrome, in which the NaCl transporter (NCC) is defective in the distal convoluted tubule (DCT). Interestingly, Kir4.1 is also relevant in this segment: along the nephron, Kir4.1 is strongly expressed in the basolateral membrane of the DCT, but it is also present in the cortical part of the thick ascending limb (CTAL) in humans and some mouse strains (Reichold et al., 2010) and in the principal cells of connecting tubule (CT) and collecting duct (CD; Ito et al., 1996; Lourdel et al., 2002; Lachheb et al., 2008; Bockenhauer et al., 2009; Reichold et al., 2010; Zhang et al., 2015). There, Kir4.1 presumably forms mainly heteromeric channels together with Kir5.1 and substantially contributes to the basolateral K^+^ conductance of these segments (Lourdel et al., 2002). Intact basolateral K^+^ conductance is essential for the establishment of hyperpolarized membrane voltage, drives other transport systems, and acts as a sensor for plasma K^+^ (see section “Kir4.1/Kir5.1: A Sensor of Plasma K^+^ in the Distal Convoluted Tubule”). Figure 4 illustrates the function of Kir4.1/Kir5.1 in the DCT.

### Urinary Loss of Na^+^ and K^+^

What is the mechanistic link between Kir4.1 inactivation in the distal nephron and electrolyte disturbance in EAST/SeSAME syndrome? Inactivation of Kir4.1 leads to a drastic reduction in basolateral K^+^ conductance and depolarization, because Kir5.1 alone cannot form functional channels. Thus, tubular transport is directly affected: the pump–leak mechanism described by Koefoed-Johnsen and Ussing (1958)—the coupling of Na^+^/K^+^-ATPase activity to the activity of basolateral K^+^ channels—is impaired, reducing the export of Na^+^. In addition, potential-dependent transport processes for efflux of Mg^2+^ and Ca^2+^ are reduced, as is the efflux of Cl^−^. Decreased salt reabsorption leads to salt loss with urine, reduction in extracellular volume, and compensatory increases in renin and aldosterone. The increase in aldosterone leads to increased Na^+^ reabsorption *via* epithelial Na^+^ channels (ENaC) in the distal nephron and partially compensates the salt loss with urine. At the same time, high aldosterone causes increased K^+^ secretion, which contributes to the development of hypokalemia. The “silencing” of DCT-mediated transport also leads to morphological changes as indicated by a flattened epithelium and reduced density of mitochondria, a situation reminiscent of tubular atrophy in Gitelman syndrome (Loffing et al., 2004; Reichold et al., 2010).

### Kir4.1/Kir5.1: A Sensor of Plasma K^+^ in the Distal Convoluted Tubule

**Figure 5:**
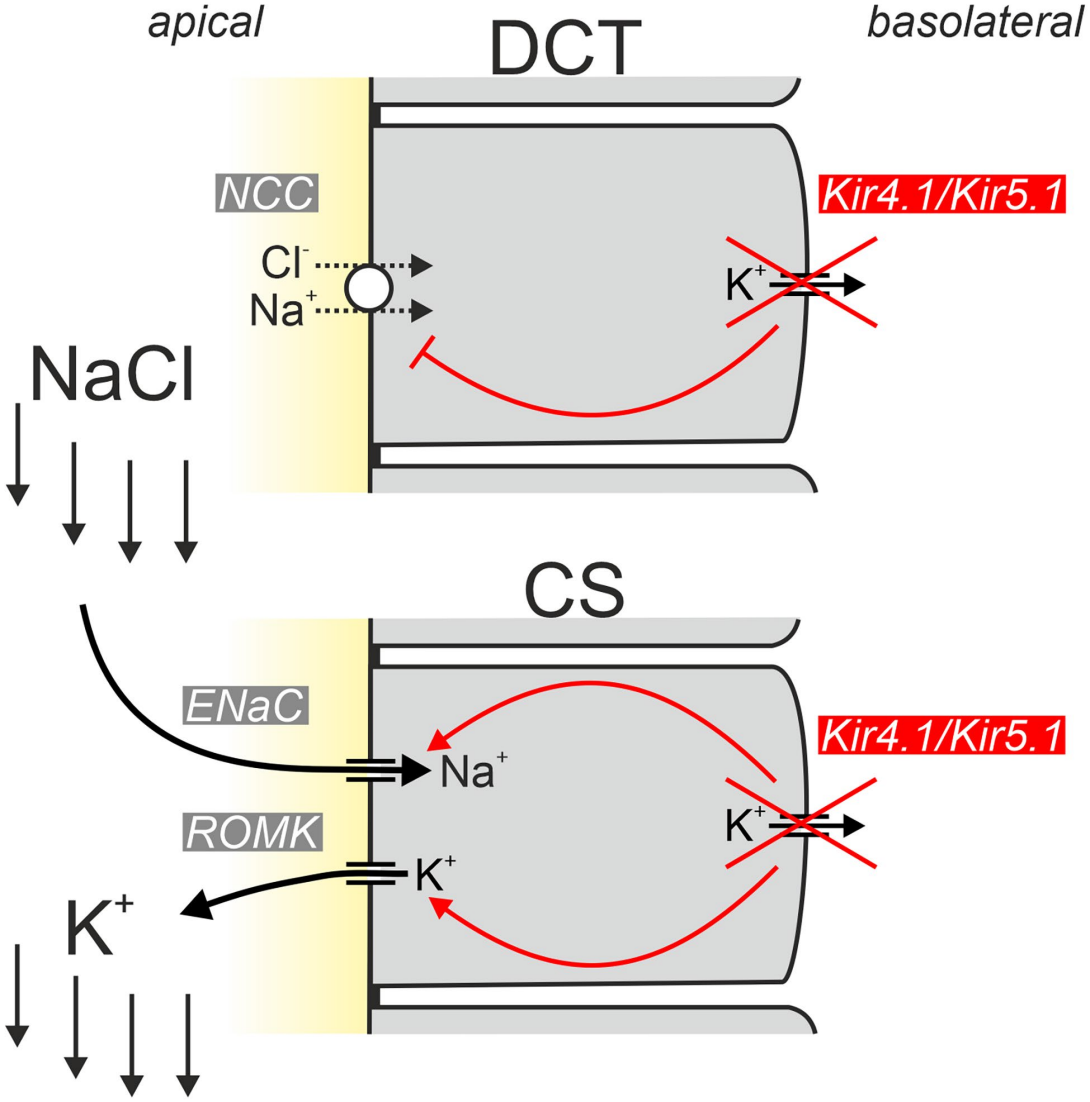
Simplified model explaining the hypokalemic phenotype of EAST/SeSAME patients. Inactivation of basolateral Kir4.1 in the distal convoluted tubule (DCT) results in reduced potential-driven transport across the basolateral membrane and inhibition of apical NCC-mediated uptake of NaCl. As a consequence, increased amounts of NaCl enter the collecting system (CS), where Na^+^ is taken up by ENaC and K^+^ is secreted by ROMK channels leading to urinary K^+^ loss. Inactivation of Kir4.1 in the CS worsens urinary K^+^ loss since apical ENaC and ROMK activities are unleashed and the physiological inhibition (*via* Kir4.1-dependent signaling) of both conductances under hypokalemic conditions is abrogated [modified from Penton et al. (2020)].

In addition to this “classical” aldosterone-induced K^+^ loss, another aspect likely contributes to the hypokalemia in EAST/SeSAME syndrome: Sorensen and colleagues found that NaCl reabsorption by the thiazide-sensitive NaCl transporter (NCC) is completely put at the service of K^+^ excretion under certain circumstances: in hyperkalemia, the increased K^+^ concentration is sensed by DCT cells and leads to inhibition of NCC (Sorensen et al., 2013). In this way, more NaCl enters the collecting system (CS) consisting of connecting tubule (CT) and collecting duct (CD), where Na^+^ is taken up *via* ENaC in principal cells. Na^+^ uptake depolarizes the apical membrane, thereby maximizing K^+^ secretion through K^+^ channels in these cells. *Via* this inter-segmental functional crosstalk, K^+^-mediated inhibition of NCC ensures that Na^+^ availability in CS does not limit the extent of K^+^ secretion during hyperkalemia. How is this related to the hypokalemia in EAST/SeSAME syndrome? Solid experimental evidence indicates that Kir4.1/Kir5.1 channels are indispensable for the proper “plasma K^+^ sensing” of DCT cells and inactivation of Kir4.1 leads to impairment of Na^+^ reabsorption similar to the effect of hyperkalemia (Cuevas et al., 2017; Wang et al., 2018). Kir4.1 is considered an essential component for plasma K^+^ sensing. Physiologically, hyperkalemia reduces K^+^ efflux through basolateral Kir4.1/Kir5.1, depolarizes DCT cells and thereby reduces Cl^−^ efflux. Increased intracellular Cl^−^ then appears to inhibit WNK kinases, ultimately leading to decreased phosphorylation of NCC and its inhibition (Terker et al., 2015; Wang et al., 2018). Inactivation of Kir4.1 likely causes a situation with depolarization and increased intracellular Cl^−^ similar to hyperkalemia and prevents the phosphorylation of NCC that is required for NCC activity. Thus, in EAST/SeSAME patients, the loss of Kir4.1 probably impairs transport across both the apical and the basolateral membrane (Figure 5).

It is widely accepted that impairment of the DCT is crucial for the development of tubulopathy in EAST/SeSAME syndrome. But is this focus on the DCT justified, given that Kir4.1 is expressed not only in the DCT but also the cTAL and in particular in the principal cells of the connecting tubule and collecting tubule? Penton and colleagues addressed this question by selectively inactivating Kir4.1 in the CS (Penton et al., 2020). Under control conditions, CS-specific Kir4.1 knockout mice were without obvious phenotype. However, when animals were put on a low K^+^ diet, the knockout mice developed marked hypokalemia. These data indicate that Kir4.1 is not only important for K^+^ sensing in the DCT but is also part of a K^+^ sensor in the CS and helps to adapt K^+^ secretion there to plasma K^+^ concentration. This adaptation no longer functions in CS-specific Kir4.1 knockout mice, and the animals show increased ENaC and ROMK activity under hypokalemia (Su et al., 2016; Penton et al., 2020). The exact signaling pathways modulated by Kir4.1 in the CS are currently unknown. It is conceivable that the WNK1 and mTORC2-dependent regulation of apical K^+^ secretion by ROMK may be influenced by plasma K^+^ *via* a K^+^-sensing mechanisms involving basolateral Kir4.1/Kir5.1.

In summary, the disturbed Na^+^ and K^+^ handling in EAST/SeSAME syndrome thus results from defects in at least two nephron segments (Wang et al., 2018; Zhang et al., 2018, 2021; Wu et al., 2019a,b): (i) a major consequence of the Kir4.1 defect is the impaired function of the DCT, which leads to flooding of the CS with NaCl due to decreased NCC-mediated NaCl reabsorption. Due to the large amount of Na^+^ and the increased aldosterone, Na^+^ reabsorption is increased in the CS and, in parallel, K^+^ secretion is enhanced; (ii) in EAST/SeSAME syndrome, the inadequately high K^+^ secretion is worsened by a dysregulation of the CS itself, which is no longer able to properly adapt the apical transport of Na^+^ and K^+^ to dietary Na^+^ and K^+^ intake due to the lack of functioning Kir4.1 channels.

### Impaired Handling of Mg^2+^ and Ca^2+^

Typically, EAST/SeSAME patients exhibit hypermagnesiuria and hypocalciuria, as is also characteristic of other situations with impaired DCT function (e.g., Gitelman syndrome, chronic hydrochlorothiazide treatment [Schultheis et al., 1998; Nijenhuis et al., 2005)]. The current understanding of the pathophysiology underlying these symptoms is discussed below.

Transcellular reabsorption of Mg^2+^ is restricted to the distal convoluted tubule (DCT). At the apical membrane, TRPM6 (transient receptor potential melastatin, subtype 6) channels, supported by TRPM7 in its surface expression, allow Mg^2+^ influx into the DCT cell (Schlingmann et al., 2002; Chubanov et al., 2004; Voets et al., 2004). As basolateral extrusion mechanisms, Na^+^/Mg^2+^ exchangers and an Mg^2+^-ATPase have been proposed (Dai et al., 2001; Ellison et al., 2021). How does decreased Mg^2+^ reabsorption occur in EAST/SeSAME syndrome? The exact mechanisms underlying this are the subject of debate (Bandulik et al., 2011; Claverie-Martin et al., 2021). Interestingly, reduced NaCl transport, as also seen in thiazide treatment or Gitelman syndrome, leads to shortening of the DCT segment and downregulation of the Mg^2+^ influx pathway (Maeoka and McCormick, 2020; Franken et al., 2021). This linkage of NaCl and Mg^2+^ transport may also be present in EAST/SeSAME syndrome, where NaCl transport in the DCT is also severely decreased and DCT cells are flattened and have less mitochondria (Reichold et al., 2010). Another possible mechanism is based on the membrane voltage dependence of basolateral Mg^2+^ extrusion: in patients with mutations of Kir4.1, reduction of K^+^ conductance results in depolarization of the basolateral membrane and thus the driving force for voltage-dependent Na^+^-coupled Mg^2+^ export in DCT is diminished.

Based on the changes in Mg^2+^ reabsorption, one would also expect a loss of Ca^2+^ in the urine. However, this is not the case: decreased DCT function is usually accompanied by increased reabsorption of Ca^2+^. It is thought that the most likely reason for this surprisingly increased reabsorption is increased Ca^2+^ reabsorption in the proximal tubule: hypovolemia triggered by DCT dysfunction leads to a compensatory increase in proximal tubular Na^+^ reabsorption and concomitantly to increased paracellular Ca^2+^ reabsorption in the proximal tubule (Nijenhuis et al., 2005). This is not possible for Mg^2+^, since paracellular Mg^2+^ reabsorption in the proximal tubule is low (Lelievre-Pegorier et al., 1983), resulting in hypermagnesiuria with concomitant hypocalciuria.

### Alkalosis in EAST/SeSAME Patients

As in Gitelman syndrome, patients with EAST/SeSAME syndrome show renal Na^+^ and K^+^ loss, activation of the renin–angiotensin–aldosterone system, and metabolic alkalosis. What are the mechanisms leading to alkalosis? First, the decreased reabsorption capacity of the distal convoluted tubule (DCT) in the EAST/SeSAME syndrome is likely to lead to an increase in Na^+^ reabsorption in the proximal tubule, similar to that observed with thiazide administration (Nijenhuis et al., 2005). Thiazides lead to an upregulation of NHE3-dependent Na^+^ reabsorption (Nijenhuis et al., 2005; NHE3 is an apical Na^+^-H^+^ exchanger) and thus proximal tubular bicarbonate reabsorption is likely to be enhanced, too. Furthermore, increased aldosterone leads to activation of H^+^ secretion in type A intercalated cells in the collecting duct (Wagner, 2014). Proton secretion is further facilitated by the lumen-negative transepithelial potential, which was increased by aldosterone-induced activation of ENaC channels in principal cells of the collecting duct. Although aldosterone-mediated activation of pendrin and thus bicarbonate secretion in type B intercalated cells also occurs (Ayuzawa et al., 2020; Pham et al., 2020), acid excretion ultimately predominates and causes metabolic alkalosis.

### Functional and Biophysical Consequences of Mutations in Kir4.1

**Figure 6:**
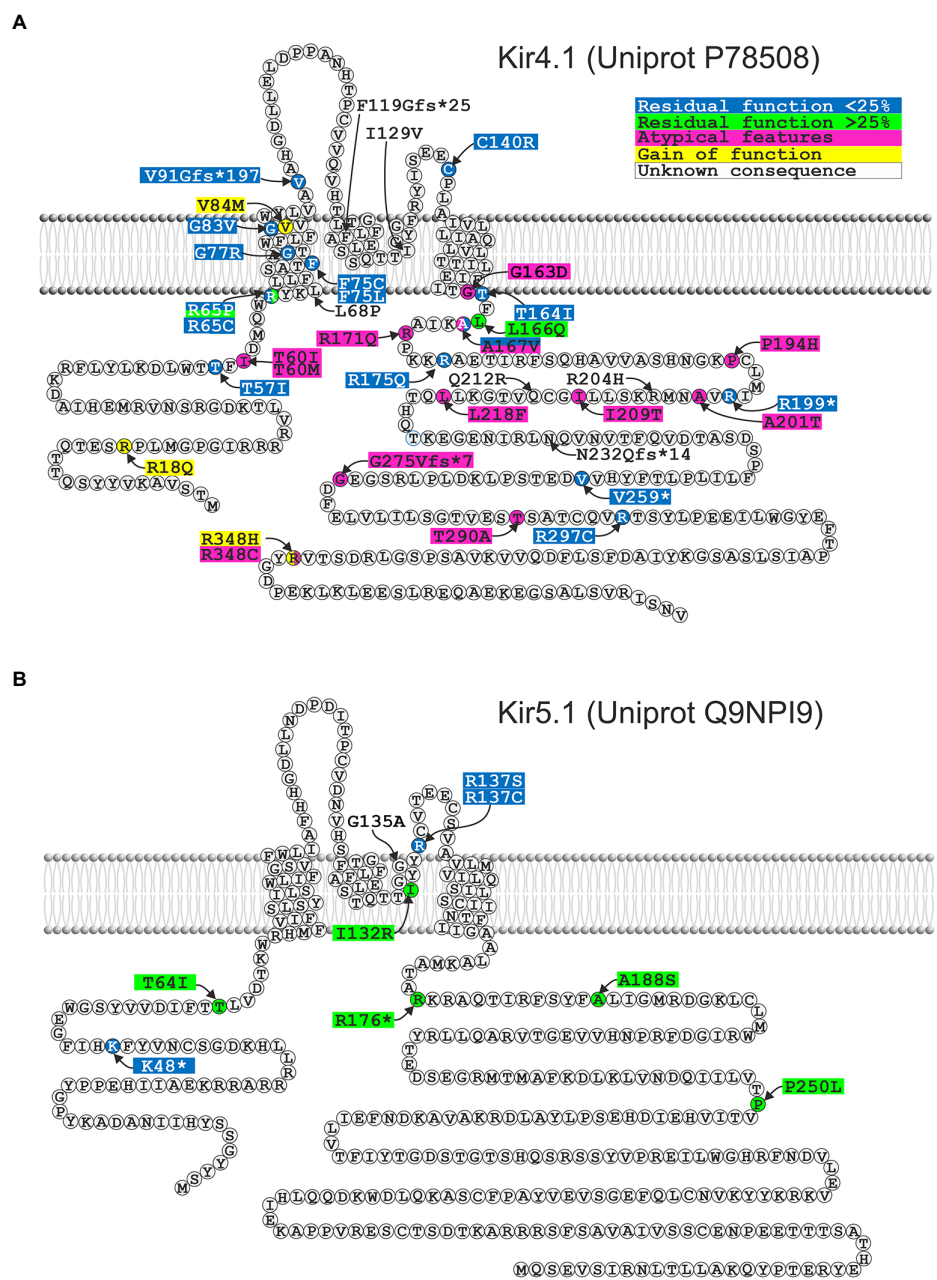
Localization of disease-associated mutations in human Kir4.1 **(A)** and Kir5.1 **(B)**. Functional consequences of Kir5.1 mutations were determined by coexpression with Kir4.1. Please note that functional deficits were more pronounced when Kir5.1 was coexpressed with Kir4.2 (Schlingmann et al., 2021; Neubauer et al., 2022). For more detailed information see Tables 1 and 2.

Table 1 lists mutations in Kir4.1 (*KCNJ10*) found by an extensive literature research and classifies them according to their functional properties, both expressed alone, or as heteromers with Kir5.1 (*KCNJ16*), or wild-type Kir4.1 if available, mimicking the heterozygous state. The majority lead to pronounced loss of function, mostly exceeding 75% loss of function, and cause the cardinal symptoms of EAST/SeSAME syndrome: epilepsy, ataxia, sensorineural hearing loss, and renal tubulopathy. We include white matter abnormalities in the table as this may be an emerging feature which is not always obvious in childhood, but seems to be present at least in some older patients (Bockenhauer et al., 2009; Scholl et al., 2009; Cross et al., 2013). A possible disease mechanism is discussed in section “Is There Structural Change to the CNS? The MLC1 Connection in Oligodendrocytes.” There are a few exceptions: Sicca et al. described a quite striking 2- to 3-fold gain-of-function in mutant Kir4.1 identified in heterozygous state in patients with autism spectrum disorders and epilepsy (Sicca et al., 2011). They identified a range of mechanisms, including increased surface expression and open probability. Only three gain-of-function mutations have been described so far (colored yellow in Table 1, Figure 6, and Supplementary Figure S1; Sicca et al., 2016). They retain some gain of function when coexpressed with wild type (Sicca et al., 2011). How both loss and gain of function in the same K^+^ channel can cause epilepsy is currently unclear.

Dominant-negative effects of a single mutated subunit on tetrameric channels are common in the voltage-gated KCNQ channel family, for example, for KCNQ1 (Wollnik et al., 1997) and other members of this family (Biervert et al., 1998; Kubisch et al., 1999; Lehman et al., 2017). Kv channel mutations also display dominant-negative effects: Some mutations in the tetrameric Kv1.1 cause dominant episodic ataxia (Adelman et al., 1995; Zerr et al., 1998). If a single mutated subunit is able to disrupt function of the tetramer completely, assuming random mutant/wild-type assembly, the conductance would be reduced to just 6.25%. Those Kir4.1 mutations which provide more than 20% function (R65P/C), yet cause typical EAST syndrome, suggest that strongly dominant-negative mutations are yet to be identified. The absence of dominant-negative mutations in Kir4.1 could reflect preferential assembly of functional, correctly folded Kir4.1 channels, or early embryonic lethality induced by dominant mutations. In addition, not all mutations have been rigorously assessed for dominant effects. The recessive nature of diseases associated with most (known) mutations is a strong argument against a significant dominant-negative effect.

Another interesting observation is the apparent lack of some typical EAST/SeSAME phenotypes in patients carrying disruptive mutations: although Kir4.1^A167V^ was initially assumed to be pathogenic when present in the context of another, more disruptive mutation on the other allele (Scholl et al., 2009), we found this mutation in homozygous state in two boys who appear to have mild, or no neurological symptoms at all, but show a typical, Gitelman-like renal phenotype (Parrock et al., 2013). Kir4.1^A167V^ showed 65% residual function (which is not expected to cause disease), but coexpression with Kir5.1 abolished function completely (Parrock et al., 2013). This could explain the lack of neurological symptoms, as only the kidney shows a complete co-localization of these two subunits. Conversely, no kidney involvement has been found in two patients with a Kir4.1 mutation affecting isoleucine 60 (I60T and I60M; Al Dhaibani et al., 2018; Nicita et al., 2018), suggesting that in this case, coexpression with Kir5.1 rescues the functional defect. These unusual mutations are colored in pink. To facilitate functional prediction of mutations identified by future work, we mapped all point and missense/truncation mutations on a simple topology model (Figure 6) as well as the point mutations on a 3D structure obtained with AlphaFold Multimer (Jumper et al., 2021), Evans and O’Neill, https://www.biorxiv.org/content/10.1101/2021.10.04.463034v1 (Supplementary Figure S1).

## Kir5.1-Linked Channelopathy

### Lessons From Kir5.1 Knockout Mice

The fact that Kir4.1 (KCNJ10) and Kir5.1 (KCNJ16) function as heteromers in the distal convoluted tubule (DCT; Lourdel et al., 2002) suggested that the effects of loss-of-function mutations in Kir5.1 would be similar to those in Kir4.1 found in EAST/SeSAME syndrome. Surprisingly, however, Teulon and colleagues (Paulais et al., 2011) had found in a Kir5.1 knockout mouse that although the animals also had hypokalemia, their remaining phenotypes were opposite to EAST/SeSAME syndrome: instead of alkalosis, metabolic acidosis was found, and instead of hypocalciuria, hypercalciuria was observed. Neurologic abnormalities or deafness were not reported in this study. The authors further examined renal function and found that the DCT (as measured by thiazide effect size) showed an increase in function, rather than a decrease as in EAST/SeSAME syndrome. Patch-clamp studies suggested that Kir4.1 in the DCT was disinhibited by inactivation of its partner Kir5.1, and the K^+^ conductance was increased. Consistent with these data, inhibition of transport in the DCT by administration of hydrochlorothiazide resulted in a stronger effect in Kir5.1 knockout animals. This elegant study therefore suggested that Kir5.1 appears to have a regulatory effect on Kir4.1 in the DCT and that omission of Kir5.1 disrupts the regulation of the K^+^ conductance in this segment. However, how does hypokalemia arise when, unlike in the EAST/SeSAME syndrome, there is increased function in the DCT? Teulon and colleagues speculated that the hypokalemia is most likely to have a cause outside the DCT and that the low urine pH and low renal ammonium excretion are indicative of a functional defect in the proximal tubule, where Kir5.1 is also expressed (Paulais et al., 2011).

### Clinical Presentation of Patients Carrying Kir5.1 Mutations

The complex phenotype of Kir5.1 knockout mice supported the view that Kir5.1 mutations could also be relevant in human disease. In 2021, two other studies independently described the effects of biallelic Kir5.1 mutations in humans (Schlingmann et al., 2021; Webb et al., 2021). The phenotype of these patients showed parallels to the symptoms described in Kir5.1 knockout mice: patients presented with a distinct renal transport dysfunction (tubulopathy), metabolic acidosis of variable severity (in fact, one patient presented with alkalosis), and sensorineural hearing loss. Interestingly, however, no CNS symptoms were observed, whereas epilepsy and ataxia are characteristic symptoms in EAST/SeSAME syndrome.

How can different symptoms be explained in patients with biallelic Kir5.1 mutations and in patients with EAST/SeSAME syndrome? (i) Kir4.1 and Kir5.1 serve different roles in the heteromeric Kir4.1/Kir5.1 channel complex: compared to homomeric Kir4.1 channels, the heteromeric Kir4.1/Kir5.1 channel is more sensitive to intracellular pH changes, the single channel conductance is increased, and inward rectification is more pronounced. Moreover, patch-clamp data from DCT cells of Kir5.1 knockout mice suggest that Kir4.1 is disinhibited in the absence of Kir5.1, and K^+^ conductance increases (Paulais et al., 2011). (ii) The expression of Kir4.1 and Kir5.1 in different tissues is overlapping but not identical (Figure 2). (iii) Kir5.1 forms more than one type of heteromeric channel: the importance of Kir5.1 as a regulatory partner of Kir4.1 in the heteromeric channel complex is well-established. However, Kir4.1 is not the only channel with which Kir5.1 interacts. For example, Kir5.1 is thought to form heteromeric channels with Kir4.2 in the proximal tubule of the kidney and in parietal cells of the stomach. Also, Kir4.1 and Kir5.1 show a different expression pattern in the inner ear, but inactivation of both channels results in impaired hearing. In the following, the pathophysiology of patients with biallelic Kir5.1 mutations is presented in more detail.

### The Peculiar Renal Phenotype in Kir5.1 Inactivation

The renal phenotype in patients with biallelic Kir5.1 mutations and in Kir5.1 knockout mice has peculiarities. Whereas in Na^+^ transport defects in the distal nephron, hypokalemia usually indicates compensatory activation of the renin–angiotensin–aldosterone system and is associated with alkalosis, the homozygous Kir5.1 defect also leads to hypokalemia, but mostly in association with acidosis. In an acid load test, patients with mutations in Kir5.1 show preserved ability to acidify urine, but increased urinary ammonium excretion is absent. What is the best explanation for the unusual combination of symptoms: hypokalemia and acidosis? As mentioned above, mutations of distal tubular proteins involved in salt transport, for example, in Bartter and Gitelman syndromes, typically lead to hypokalemia and alkalosis. In these disorders, compensatory elevated aldosterone results in Na^+^ reabsorption and K^+^ secretion in the principal cells of the collecting system and increased proton secretion in the intercalated cells. In contrast to these transport proteins, Kir5.1 expression is not restricted to the distal nephron, but Kir5.1 is probably involved with Kir4.2 in the formation of basolateral K^+^ channels of the proximal tubule. Interestingly, Kir4.2 knockout mice showed a phenotype that parallels the one seen with Kir5.1 inactivation (Bignon et al., 2020): Kir4.2 knockout mice exhibited metabolic acidosis and decreased proximal tubular ammonium formation and excretion. The proximal tubule cells were depolarized, but there was no general dysfunction of the proximal tubule; rather, the transport defect was limited to bicarbonate reabsorption and ammonium excretion. The clinical symptoms of Kir5.1 patients and functional analysis of mutant proteins indicate that loss-of-function mutations of Kir5.1 also profoundly disrupt the function of heteromeric Kir4.2/Kir5.1 channels. Indeed, data from expression systems suggest that the functional impairment caused by Kir5.1 mutations is more pronounced in Kir4.2/Kir5.1 heteromers than in Kir4.1/Kir5.1 channels. The variable extent of acidosis and of a salt-wasting phenotype in patients with Kir5.1 mutations (Schlingmann et al., 2021) may suggest that the nature of the mutation and the ability to compensate influence the degree of impairment of proximal tubular function.

### Hearing Impairment Associated With Kir5.1 Inactivation

In addition to hypokalemia and acidosis of variable severity, patients with Kir5.1 mutation had in common sensorineural hearing loss that appeared in childhood or adolescence (Schlingmann et al., 2021). The hearing loss affected mainly the higher frequencies, similar to the hearing loss in EAST/SeSAME syndrome. Interestingly, however, the expression pattern of Kir4.1 and Kir5.1 differs in the inner ear (https://umgear.org/; Burns et al., 2015). Kir4.1 is expressed primarily in intermediate cells of the stria vascularis and is responsible for endolymph composition and endocochlear potential (Hibino et al., 1997). Kir5.1 is expressed mainly in the root cells and spindle cells of the spiral ligament (Hibino et al., 2004b), where it possibly forms functional channels with Kir4.2 (Burns et al., 2015). However, unlike Kir4.1, the functional relationships and pathophysiological consequences of Kir5.1 are less understood (Hibino and Kurachi, 2006). In an elegant study, Hibino and colleagues (Hibino et al., 2004b) investigated the age dependence of Kir5.1 expression and, on the basis of these data, proposed that Kir5.1 is involved in K^+^ cycling and may play a role in the establishment of the endocochlear potential. Surprisingly, hearing function has been shown to be unaffected in a Kir5.1 knockout mouse model (Lv et al., 2021). This may indicate efficient compensatory mechanisms in this strain of Kir5.1 knockout mice that are not present to the same extent in humans with biallelic Kir5.1 mutations.

### Kir5.1 Variants: A Risk Factor for SIDS?

The strong pH dependence of Kir4.1/Kir5.1 channels and the presence of Kir5.1 in peripheral chemoreceptors and in the brainstem (D'Adamo et al., 2011) prompted Trapp and colleagues (Trapp et al., 2011) to investigate the respiratory regulation in these acidotic Kir5.1 knockout mice. Indeed, they observed a reduced ventilatory response of knockout animals to hypoxia and hypercapnia with preserved function of chemosensitivity of carotid bodies: the peripheral chemoreceptors. The authors concluded that Kir4.1/Kir5.1 channels are not an indispensable component of respiratory regulation but that they rather mildly modify the ventilatory response to stimuli.

Interestingly, Neubauer et al. (2022) recently found single nucleotide polymorphisms in the gene for Kir5.1 (KCNJ16 variants p.R137S and p.A188S) in two cases in a study of 155 cases of sudden infant death syndrome (SIDS) using exome sequencing. The authors speculated that these monoallelic variants, which have a high CADD score (Table 2), may impair central CO_2_ sensitivity and thus may have contributed to the cause of death. Surprisingly, in a patch-clamp analysis on cells coexpressing the Kir5.1 mutants with Kir4.1, only the Kir5.1 variant R137S (rs766250689) showed functional impairment, whereas the variant A188S was functional. However, it cannot be excluded that the mutant A188S also exhibits defects in a context-dependent manner, for example, in heteromer formation with other Kir channels or with respect to regulation. It is therefore conceivable that missense variants in the gene of Kir5.1, which appears to contribute to respiratory chemoreception, could play a role in a minority of SIDS cases as it has been postulated for other genes controlling respiration (Laer et al., 2015). Additional studies are needed to further explore this hypothesis and its relevance to this disease with multifactorial etiology in larger cohorts.

**Table 2:** Mutations identified in *KCNJ16*/Kir5.1.

Mutation	CADD Score	Associated phenotypes				Function with Kir4.1	Function with Kir4.2	Reference
		H	A	D	SIDS			
K48*	36	Y	N	ND		ND	ND	Webb et al., 2021
T64I	23.8	Y	N	Y		Reduced by 70%	Reduced by >90%	Schlingmann et al., 2021
I132R	24.7	Y	(Y)	Y		Reduced by 74%	Reduced by >90%	Schlingmann et al., 2021
G135A	24.4	Y	(Y)	Y		ND	ND	Schlingmann et al., 2021
R137C	25.2	Y	Y	Y		Reduced by 83%	Reduced by >90%	Schlingmann et al., 2021
R137S	24.2	ND	ND	ND	Y	Reduced by 80%	ND	Neubauer et al., 2022
R176*	34	Y	(Y)	Y		Reduced by 54%	Reduced by >90%	Schlingmann et al., 2021
A188S	23.5	ND	ND	ND	Y	Increased by 10%	ND	Neubauer et al., 2022
P250L	23.4	Y	(Y)	Y		Reduced by 39%	Reduced by >90%	Schlingmann et al., 2021
		>25% Residual function						
		<25% Residual function						
		Atypical features						
		Gain of function						
		Function unknown						

Table 2 summarizes the consequences of KCNJ16 mutations. CADD score according to https://cadd.gs.washington.edu/snv (Rentzsch et al., 2019). Associated phenotypes refer to hypokalemia (H), acidosis (A), sensorineural deafness (D), and a possible association with sudden infant death syndrome (SIDS). Yes (Y); probably Yes (Y); No (N); Not examined (ND). Color code is shown for functional effects when coexpressed with Kir4.1. * indicates a “stop codon”. Note that functional consequences were generally more severe when Kir5.1 mutants were coexpressed with Kir4.2 (Schlingmann et al., 2021).

## Conclusion and Outlook

EAST/SeSAME syndrome due to mutations in *KCNJ10* (Kir4.1) was first described in 2009. Initially, epilepsy, ataxia, sensorineural hearing loss and electrolyte abnormalities due to changes in renal salt handling appeared to be constant features of this syndrome. However, the clinical picture has become considerably more granular, and so has our understanding. One of the main recently emerging complications is that heteromerization with Kir5.1 blurs the picture. Some Kir4.1 mutations may be rescued by coexpression of Kir5.1, while others, only mildly affecting homomeric Kir4.1, may become pathogenic. Different levels of residual function, or particular properties such as pH sensitivity, required in different organs, may determine which systems will show symptoms. Some phenotypes only develop over time, as degenerative changes dominate. In addition, there is considerable clinical variability for patients carrying the same mutation(s) even within one family. This may indicate that modifier genes, epigenetic differences, or even environmental factors, determine much of the severity and the range of symptoms. Recently, we described a novel syndrome due to mutations in Kir5.1. It shares some features, but none of the neurological symptoms, with EAST/SeSAME syndrome. The kidney phenotype also shows some striking differences to EAST/SeSAME, and future work will show whether this is due to interaction of Kir5.1 with Kir4.2, in which no human mutations have been described so far. We expect next-generation sequencing and genome-wide association studies to provide the clues and animal models to confirm hypotheses and fill in the remaining gaps in our understanding. In addition, efforts are underway to develop specific compounds with the intention of targeting these channels (Kharade et al., 2018; Ohno et al., 2021; Weaver and Denton, 2021). This will provide further insight into the fascinating physiology of this group of K^+^ channels and can pave the way for new therapeutic strategies.

## Author Contributions

All authors listed have made a substantial, direct, and intellectual contribution to the work and approved it for publication.

## Funding

Funded by the Deutsche Forschungsgemeinschaft (DFG, German Research Foundation), project number 387509280, SFB 1350 to RW and St. Peter’s Trust funding to AZ.

## Conflict of Interest

The authors declare that the research was conducted in the absence of any commercial or financial relationships that could be construed as a potential conflict of interest.

## Publisher’s Note

All claims expressed in this article are solely those of the authors and do not necessarily represent those of their affiliated organizations, or those of the publisher, the editors and the reviewers. Any product that may be evaluated in this article, or claim that may be made by its manufacturer, is not guaranteed or endorsed by the publisher.

## Footnotes

^1^ https://www.genenames.org/data/genegroup/#!/group/183

^2^ https://www.guidetopharmacology.org/GRAC/FamilyDisplayForward?familyId=133&familyType=IC

^3^ https://www.guidetopharmacology.org/

## Supplementary Material

Supplementary Figure S1 Stereo image (rendered with PyMol 2.5.2) of a Kir4.1/Kir5.1 model generated with AlphaFold Multimer (Jumper et al., 2021), Evans and O’Neill; https://www.biorxiv.org/content/10.1101/2021.10.04.463034v1. Kir4.1 chains are brown and Kir5.1 chains are gray. Point mutations identified in Kir4.1 and Kir5.1-associated diseases are highlighted in blue (for <25% residual function), green >25% residual function, cyan if one report found >25% and another <25% residual function, yellow (gain of function) and pink (atypical behavior, that is, absence of one or more cardinal symptoms). We truncated both N- and C-termini slightly, to reduce complexity. AlphaFold yielded three models with almost identical ranking, but only one of them conformed to the alternating subunit structure postulated by (Lagrutta et al., 1996). We also highlight mutations in Kir5.1 (described below) in the model for reference. Mutations leading to more than 75% loss of function in homomers and show typical EAST features are colored in blue, mutations with residual function in green, and mutations that show a partial clinical phenotype are colored in pink. Gain-of-function mutations are colored in yellow.
