# Supplementary figures and images for: EAST/SeSAME Syndrome and Beyond: The Spectrum of Kir4.1- and Kir5.1-Associated Channelopathies
Source: Front Physiol. 2022 Mar 15;13:852674. doi: 10.3389/fphys.2022.852674 (PMC8965613; doi:10.3389/fphys.2022.852674)

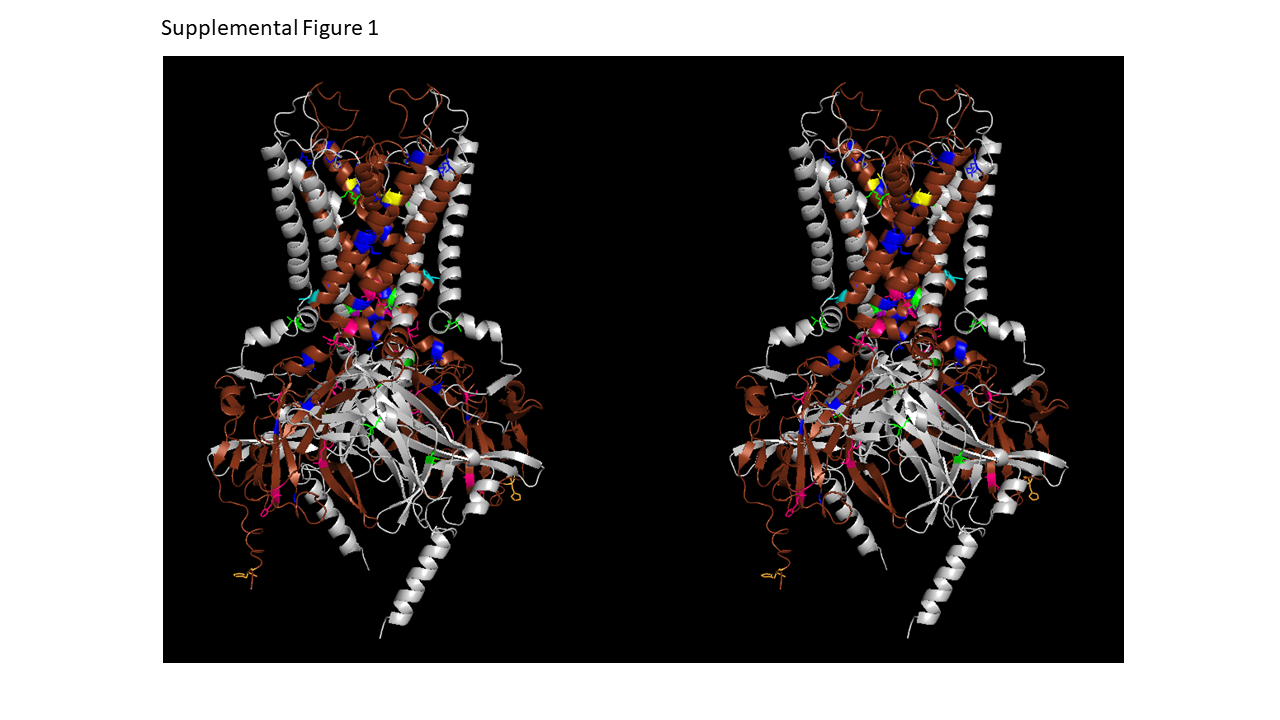

Supplement: Supplementary Figure S1 — Stereo image (rendered with PyMol 2.5.2) of a Kir4.1/Kir5.1 model generated with AlphaFold Multimer (Jumper et al., 2021), Evans and O’Neill; https://www.biorxiv.org/content/10.1101/2021.10.04.463034v1. Kir4.1 chains are brown and Kir5.1 chains are gray. Point mutations identified in Kir4.1 and Kir5.1-associated diseases are highlighted in blue (for <25% residual function), green >25% residual function, cyan if one report found >25% and another <25% residual function, yellow (gain of function) and pink (atypical behavior, that is, absence of one or more cardinal symptoms). We truncated both N- and C-termini slightly, to reduce complexity. AlphaFold yielded three models with almost identical ranking, but only one of them conformed to the alternating subunit structure postulated by (Lagrutta et al., 1996). We also highlight mutations in Kir5.1 (described below) in the model for reference. Mutations leading to more than 75% loss of function in homomers and show typical EAST features are colored in blue, mutations with residual function in green, and mutations that show a partial clinical phenotype are colored in pink. Gain-of-function mutations are colored in yellow. [file Image_1.TIF]
